# High Prevalence of Human T-Lymphotropic Virus Infection in Indigenous Women from the Peruvian Amazon

**DOI:** 10.1371/journal.pone.0073978

**Published:** 2013-09-05

**Authors:** Magaly M. Blas, Isaac E. Alva, Patricia J. García, Cesar Cárcamo, Silvia M. Montano, Nicanor Mori, Ricardo Muñante, Joseph R. Zunt

**Affiliations:** 1 Epidemiology, STD and HIV Unit, School of Public Health and Administration, Universidad Peruana Cayetano Heredia, Lima, Peru; 2 United States Naval Medical Research Unit No. 6 (NAMRU-6), Callao, Peru; 3 Ucayali Regional Health Directorate, Ministry of Health, Ucayali, Peru; 4 School of Public Health and Community Medicine, Departments of Neurology, Global Health and Medicine, University of Washington, Seattle, Washington, United States of America; Centers for Disease Control and Prevention, United States of America

## Abstract

**Background:**

In an earlier study, we detected an association between human T-cell lymphotropic virus (HTLV) infection and cervical human papillomavirus (HPV) in indigenous Amazonian Peruvian women of the Shipibo-Konibo ethnic group. As both HTLV and HPV can be transmitted sexually, we now report a population-based study examining the prevalence and risk factors for HTLV-1 and HTLV-2 infection in this population.

**Methods:**

Between July and December 2010, we conducted a comprehensive screening for HTLV among Shipibo-Konibo women 15 to 39 years of age living in two communities located in Lima and in 17 communities located within four hours by car or boat from the Amazonian city of Pucallpa in Peru.

**Results:**

We screened 1,253 Shipibo-Konibo women for HTLV infection 74 (5.9%) tested positive for HTLV-1, 47 (3.8%) for HTLV-2 infection, and 4 (0.3%) had indeterminate results. In the multivariate analysis, factors associated with HTLV-1 infection included: older age (Prevalence Ratio (PR): 1.04, 95% CI 1.00–1.08), primary education or less (PR: 2.01, 95% CI: 1.25–3.24), younger or same age most recent sex partner (PR: 1.66, 95% CI: 1.00–2.74), and having a most recent sex partner who worked at a logging camp (PR: 1.73, 95% CI: 1.09–2.75). The only factor associated with HTLV-2 infection was older age (PR: 1.08, 95% CI: 1.03–1.12).

**Conclusion:**

HTLV infection is endemic among Shipibo-Konibo women. Two characteristics of the sexual partner (younger age and labor history) were associated with infection in women. These results suggest the need for implementation of both HTLV screening during the antenatal healthcare visits of Shipibo-Konibo women, and counseling about the risk of HTLV transmission through prolonged breastfeeding in infected women. We also recommend the implementation of prevention programs to reduce sexual transmission of these viruses.

## Background

Human T-cell lymphotropic virus type 1 (HTLV-1) was the first oncogenic human retrovirus to be discovered [1]. Currently, there are three other known members of the HTLV family of viruses: HTLV-2, HTLV-3 and HTLV-4 [2]–[4]. HTLV-1 is associated with adult T-cell leukemia/lymphoma and with myelopathy/tropical spastic paraparesis [5]–[7]. HTLV-1 is also associated with tuberculosis, uveitis, infective dermatitis, and strongyloidiasis [8]–[11]. HTLV-2 infection produces less disease than HTLV-1, but has been associated with recurrent pneumonia and neuropathy [12]. It is not known if HTLV-3 or -4 are associated with disease in humans.

HTLV-1 and -2 infections are transmitted via breastfeeding, sexual contact, and parenteral transmission via blood transfusion and intravenous drug use [5], [12]. HTLV is estimated to infect 10 to 20 million people worldwide and is endemic in Japan, the Melanesian islands, the Middle East, sub-Saharan Africa, South America and the Caribbean [13]–[16]. In South America and the Caribbean, specifically in Jamaica, Martinique, Guyana, French Guyana, Colombia, and the north of Brazil, HTLV-1 infection is frequent among people of African descent, whereas in Peru and northern Argentina it is more prevalent among indigenous people [17]–[21].

In Peru, most HTLV infections have been reported in indigenous people from the Andean Region (Quechua and Aymara ethnicities), and among African-Peruvian and mestizo populations (a mix of Spanish and Andean indigenous blood) [15], [20], [22], [23]. In contrast, there is limited information about HTLV infection in Amazonian ethnic groups, with limited reports of HTLV infection in Shipibo-Konibo, Kechwa-Lamas, Wampis, and Harakmbut villages [19], [21], [24]. HTLV has also been reported in populations at high risk for sexually transmitted infections, such as female sex workers (FSW) and men who have sex with men (MSM) [25]–[27].

As part of a previous study conducted among ten indigenous groups in the Peruvian Amazon, we found 18 HTLV infections restricted to the Shipibo-Konibo people, one of the largest Peruvian Amazonian ethnic groups residing along the Ucayali river [24]. However the sample size for that study limited the exploration of risk factors for the infection. Therefore, the objective of our study was to conduct a population-based study in the Shipibo-Konibo ethnic group to determine the prevalence and risk factors for HTLV-1 and HTLV-2 infections among women 15 to 39 years of age.

## Methods

### Ethics Statement

Our study was approved by the Institutional Review Boards (IRB) of the University of Washington in Seattle, the Universidad Peruana Cayetano Heredia, and U.S. Naval Medical Research Unit No.6 (NAMRU-6) in Callao, Peru. All enrollees provided written informed consent prior to their participation in our study. If participants were at least 18 years old, or between 15 and 17 years of age and married/cohabitating for two or more years, and they agreed to participate, they were invited to participate and asked to sign the informed consent. Other consenting eligible women signed an assent and their parent or legal guardian signed an informed consent. The IRB considered participants between 15 and 17 years of age and married/cohabitating for two or more years as emancipated, not requiring parental permission for enrollment. In the context of Amazonian ethnic groups, it is common for women to become emancipated before 18 years of age, usually through marriage or cohabitation. At this age they leave their parent’s home, raise their children and provide economic support for their families.

### Study Design

As part of a study to determine the association between HTLV and cervical HPV infection, between July and December 2010 we conducted a comprehensive screening for HTLV infection among Shipibo-Konibo women 15 to 39 years old who agreed to participate and lived in any of the Shipibo-Konibo communities located in Lima or within four hours (most often within one hour) by car or boat from the city of Pucallpa. The screening was conducted through household visits. We defined women as belonging to the Shipibo-Konibo ethnic group if they had at least one of the following characteristics: 1) they self-identified as Shipibo-Konibo, 2) they spoke Shipibo-Konibo language, or 3) at least one of their parents belonged to the Shipibo-Konibo ethnic group.

### Recruitment Process

Details of the recruitment process and laboratory techniques are described elsewhere [21]. Briefly, in our study we first established community advisory boards (CABs) in Lima and Pucallpa. The objective of these CABs was to provide advice regarding approaches to engage the communities in the project and to receive feedback regarding study procedures. After receiving feedback from the CABs, we initiated visits to the communities. The study visits were divided into two phases. In the first phase, we visited the leader of each community selected for the project. If the leader agreed to participate in the study with his community, he signed the community informed consent; we then conducted a census of the community to identify eligible women. Later on, we individually approached all eligible women at their home to assess their interest in participation. Participants who were at least 18 years old, or between 15 and 17 years of age and married or cohabitating for two or more years, and willing to participate, were asked to sign the informed consent. Women who were 15 to 17 years of age but not married or cohabiting were asked to sign an assent in addition to a signed informed consent from their parent or legal guardian. Afterwards, we administered a face-to-face questionnaire in Spanish that included sections on demographics, previously identified risk factors for HTLV infection (e.g. prolonged breastfeeding, blood transfusion), risk behaviors for sexually transmitted infections (STI), information regarding most recent sexual partner and symptoms of HTLV-associated diseases. Then we took a venous blood sample to test for HTLV infection [21].

### Sample Collection and Transportation

Venous blood samples were collected using 10 ml vacuum blood collection tubes, transported in coolers and delivered within 6 hours of collection to the laboratory in Lima or Pucallpa. Serum was promptly separated, divided into aliquots in vials and frozen at −20°C. Frozen serum aliquots were sent weekly for HTLV testing from Pucallpa to the NAMRU-6 laboratory in Callao via air courier in Styrofoam coolers wrapped in metal. Serum samples obtained in Lima were sent directly to NAMRU-6 for processing and analysis.

### HTLV Testing Techniques

HTLV testing was performed at NAMRU-6 in Callao, Peru. A woman was considered to be HTLV infected if she had a positive test for HTLV ELISA (Vironostika, North Carolina), and the confirmatory Western blot assay (HTLV-1/2 Blot 2.4, Genelabs Diagnostics, Singapore) revealed bands representing Gag (p24, p19) and Env proteins (Gp46, GD21 and rgp 46-I). An individual was considered HTLV-2 seropositive if the ELISA assay was positive and confirmatory Western blot assay revealed bands representing p24, GD21 and rgp46-II. If Gag and Env proteins were absent, but other HTLV specific bands, such as p53 or p19 were present, the individual was considered indeterminate. Subjects with indeterminate results were excluded from the analysis. PCR testing was not performed since the specimens needed for this testing were not collected in the study.

### Provision of HTLV Results

Approximately two months after the first visit, we carried out a second visit to the communities to deliver HTLV test results [21]. HTLV positive participants were informed that HTLV is a lifelong infection, that it is not the AIDS virus and that it does not cause AIDS. They were also given information regarding modes of transmission. HTLV-1 positive participants were given information about potential health conditions associated with the virus and the probability of developing these conditions. HTLV-1 positive women, and particularly pregnant and breastfeeding women were given information about the increased risk of HTLV-1 transmission to their offspring if they breastfeed longer than 6 months. However, we did not provide a specific recommendation to stop breastfeeding. HTLV-2 positive participants were given information about the limited association with affecting health conditions.

### Statistical Methods

Analyses were performed using Stata (v8.0; Stata Corp., College Station, TX). To identify factors associated with HTLV-1 and HTLV-2 infections, crude and adjusted prevalence ratios (PR) and their corresponding 95% confidence intervals were estimated using generalized linear models adjusting by community of residence. Analysis of HTLV-1 and 2 infections were conducted separately because of their potential differences in risk factors. Variables independently associated with either HTLV infection at a level of significance of 0.1 were included in a multivariate model that was refined using backward stepwise selection process. Predictors significant at the 0.05 level were retained in the final model.

## Results

**Figure 1:**
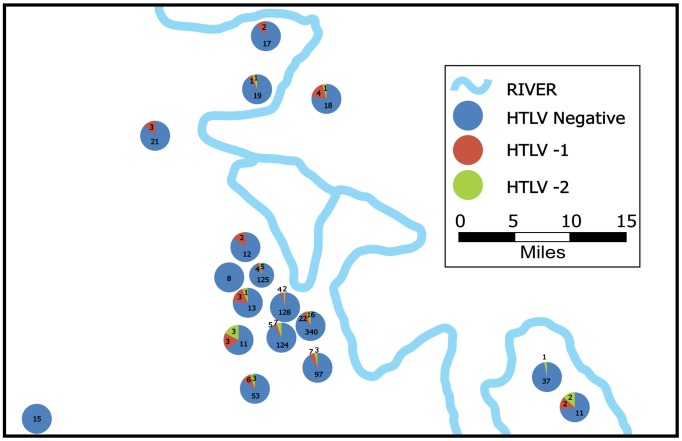
Frequency of HTLV-1 and -2 infections per community in the Ucayali region.

Between July and December 2010, we approached leaders of 21 Shipibo-Konibo communities and 19 consented participation of their communities. Two communities were located in Lima, and 17 were Amazon jungle communities located near the city of Pucallpa. In these communities we approached 1,460 women, of whom 58 refused to participate. Of the 1,402 consenting women 149 were excluded because they did not belong to the Shipibo-Konibo ethnic group. All the remaining 1,253 eligible women could speak Spanish. Of these women, 74 (5.9%) tested positive for HTLV-1, 47 (3.8%) for HTLV-2 infection, and four (0.3%) had indeterminate results. Participants with indeterminate results were excluded from further analysis. The combined HTLV-1/2 prevalence was 9.7%. There were no dual infections. HTLV infected participants were born in seven out of 10 districts included in the Ucayali region and in 4 out of 11 districts included in the Loreto region. The distribution of HTLV within communities did not follow a clear geographical pattern. Figure 1 shows the frequency of HTLV-1 and -2 positive individuals per community in the Ucayali Region. To protect the identity of the communities participating, only their relative locations are shown and the two easily identifiable communities in Lima were excluded. This is in response to the request of the IRB which agreed with the communities to never report HTLV prevalence by community.

HTLV-1 infected women were more likely than HTLV-negative participants to be older (There was a 4% increase in HTLV-1 prevalence for each additional year of age), less educated, married or cohabiting, to have a last sexual partner either younger or of the same age than HTLV negative women, to live with current sex partner, to not use a condom in their last sexual intercourse, to have a last sex partner who worked at a logging camp and to self-report a history of tuberculosis (Table 1).

**Table 1:** Factors associated with human T cell lymphotropic virus type 1 (HTLV-1) and HTLV-2 infections among women from the Shipibo-Konibo ethnic group.

Variable	HTLV-1 Positive/Tested (%)	PR (95% CI)	HTLV-2 Positive/Tested (%)	PR (95% CI)
**Age, years (mean, SD)**	28.2 (7.16)	**1.06 (1.04–1.08)**	28.9 (6.49)	**1.08 (1.05–1.10)**
**Education**				
More than primary education	42/899 (4.7)	1.0	35/892 (3.9)	1.0
Primary education or less	32/303 (10.6)	**2.26 (1.51–3.39)**	12/283 (4.2)	1.11 (0.66–1.86)
**Has migrated from her community**				
No	21/400 (5.3)	1.0	8/387 (2.1)	1.0
Yes	53/802 (6.6)	1.26 (0.71–2.22)	39/788 (5.0)	**2.73 (1.15–6.46)**
**Marital status**				
Single/Separated/Widower	15/385 (3.9)	1.0	12/382 (3.1)	1.0
Married/Cohabitating	59/817 (7.2)	**1.85 (1.15–2.98)**	35/793 (4.4)	1.37 (0.94–1.99)
**Sexually active**				
No	3/95 (3.2)	1.0	1/93 (1.1)	1.0
Yes	71/1,101 (6.5)	2.04 (0.84–4.96)	46/1,076 (4.3)	**3.89 (1.31–11.57)**
**Age at first sexual intercourse (mean, SD)**	14.8 (1.33)	0.91 (0.81–1.02)	14.8 (1.69)	0.93 (0.79–1.08)
**Age at first pregnancy, mean (range)**	16.6 (12–25)	0.91 (0.81–1.02)	17.1 (12–22)	0.92 (0.76–1.11)
**New sexual partner within the last 12 months**				
No	65/979 (6.6)	1.0	40/954 (4.2)	1.0
Yes	6/117 (5.1)	0.77 (0.31–1.91)	6/117 (5.1)	1.25 (0.60–2.62)
**Have previously worked at a logging camp**				
No	70/1,157 (6.1)	1.0	45/1,132 (4.0)	1.0
Yes	4/45 (8.9)	1.47 (0.56–3.89)	2/43 (4.7)	1.20 (0.46–3.12)
**Characteristics of the last sex partner**				
Stable last sex partner	67/1,020 (6.6)	1.0	42/995 (4.2)	1.0
Non-stable last sex partner	4/81 (4.9)	0.75 (0.27–2.13)	4/81 (4.9)	1.17 (0.48–2.84)
**Age of most recent sex partner**				
Current sex partner older	51/898 (5.7)	1.0	32/879 (3.6)	1.0
Current sex partner younger or same age	20/200 (10.0)	**1.76 (1.22–2.55)**	14/194 (7.2)	**2.04 (1.00–3.88)**
**Lives with current sex partner**				
No	14/318 (4.4)	1.0	10/314 (3.2)	1.0
Yes	57/780 (7.3)	**1.66 (1.03–2.68**)	36/759 (4.7)	1.45 (0.86–2.45)
**Knows that partner had another partner**				
No	15/241 (6.2)	1.0	7/233 (3.0)	1.0
Yes	13/310 (4.2)	0.67 (0.34–1.34)	19/316 (6.0)	2.00 (0.83–4.81)
Didn’t know	43/545 (7.9)	1.27 (0.69–2.34)	20/522 (3.8)	1.21 (0.60–2.47)
**Used condom at last sexual intercourse**				
No	71/970 (7.3)	1.0	43/942 (4.6)	1.0
Yes	0/126 (0.0)	**0.00 (p<0.001)** *	3/129 (2.3)	0.52 (0.20–1.34)
**Partner belongs to the Shipibo-Konibo ethnic group**				
No	13/220 (5.9)	1.0	8/215 (3.7)	1.0
Yes	57/873 (6.5)	1.10 (0.58–2.12)	38/854 (4.5)	1.17 (0.44–3.08)
**Most recent sex partner worked at a logging camp**				
No	43/800 (5.4)	1.0	33/790 (4.2)	1.0
Yes	26/247 (10.5)	**1.96 (1.35–2.85)**	11/232 (4.7)	1.04 (0.43–2.48)
**Was breastfeed by a person other than her mother**				
No	37/689 (5.4)	1.0	24/676 (3.6)	1.0
Yes	37/513 (7.2)	1.34 (0.88–2.06)	23/499 (4.6)	1.35 (0.86–2.12)
**Had ever received blood transfusion**				
No	73/1186 (6.2)	1.0	46/1,159 (4.0)	1.0
Yes	1/16 (6.3)	1.02 (0.13–7.83)	1/16 (6.3)	1.61 (0.38–6.84)
**Had bleeding during a dental procedure**				
No	74/1,199 (6.2)	1.0	46/1,171 (3.9)	1.0
Yes	0/3 (0.00)	0.00 (p = 1.00)*	1/4 (25.0)	**6.50 (1.12–37.62)**
**Family history of leukemia or lymphoma**				
No	69/1,125 (6.1)	1.0	45/1,101 (4.1)	1.0
Yes	2/30 (6.7)	1.09 (0.23–5.18)	2/30 (6.7)	1.67 (0.61–4.54)
**Family history of Tropical Spastic Paraparesis**				
No	67/1092 (6.1)	1.0	45/1070 (4.2)	1.0
Yes	2/21 (9.5)	1.55 (0.32–7.62)	0/19 (0.0)	0.00 (p = 1.00)*
**Ever had crusted scabies for more than a month**				
No	69/1,163 (5.9)	1.0	42/1,136 (3.7)	1.0
Yes	5/36 (13.9)	2.34 (0.60–9.10)	5/36 (13.9)	**3.09 (1.10–8.71)**
**History of TBC**				
No	69/1,176 (5.9)	1.0	45/1,152 (3.9)	1.0
Yes	5/26 (19.2)	**3.28 (1.61–6.66)**	2/23 (8.7)	2.27 (0.87–5.97)

* Confidence interval cannot be calculated.

HTLV-2 infected women were more likely to be older and not living in the community where they were born. They were also more likely to be sexually active, to have a last sexual partner younger or of the same age than HTLV negative women, to report a history of bleeding during a dental procedure and a history of crusted scabies (Table 1).

We did not find an association between having had a partner who used intravenous drug use, having tattoos, piercing or surgery and HTLV-1 or -2 infection (data not shown). We were not able to evaluate duration of breastfeeding as a risk factor for HTLV-1 or -2 infection, since 850 (68.1%) of participants did not recall this information. When analyzing women who knew the duration of breastfeeding, the mean duration of breastfeeding was similar for both HTLV seropositive and seronegative participants (15.3 months vs. 15.1 months, p = 0.85).

Overall, 15.0% (17) participants who reported they were pregnant at the time of the interview were infected with HTLV, 10 with HTLV-1 and 7 with HTLV-2. The mean number of sexual partners during the last 12 months among HTLV negative, HTLV-1 and HTLV-2 positive participants was similar (1.0 vs. 1.0 vs. 1.2 partners). Among sexually active participants, the combined HTLV-1 and -2 prevalence was 10.2%, whereas among participants reporting not having initiated sexual activity the prevalence was 4.2%, suggesting vertical transmission is a common cause of HTLV-1 and -2 infection.

In the multivariate analysis, factors associated with HTLV-1 infection were age, having primary education or less, having the most recent sex partner either younger or of the same age, and having the most recent sex partner who worked at a logging camp. The only factor independently associated with HTLV-2 infection was age (Table 2).

**Table 2:** Multivariate analysis of factors associated with human T cell lymphotropic virus type 1 (HTLV-1) and HTLV-2 infections in women from the Shipibo-Konibo ethnic group.

Model	Adjusted Prevalence ratios (95% CI)
**Model for HTLV-1 infection**	
Age	1.04 (1.00–1.08)
Primary education or less	2.01 (1.25–3.24)
Last sex partner younger or same age	1.66 (1.00–2.74)
Last sex partner worked at a logging camp	1.73 (1.09–2.75)
**Model for HTLV-2 infection**	
Age	1.08 (1.03–1.12)

## Discussion

To our knowledge, this is the largest population-based study to assess the prevalence of HTLV-1 and -2 infections in indigenous populations in Latin America. We found a combined HTLV-1 and -2 prevalence of 9.7% among Shipibo-Konibo women, the highest prevalence described in an indigenous group in Peru.

In the multivariate analysis, older age was associated with both HTLV-1 and -2 infections. Other studies that have assessed risk factors for HTLV infection have reported that seroprevalence increases with age, suggesting that sexual intercourse plays an important role in the transmission of this virus [28], [29], or suggesting a cohort effect for an infection that is diminishing. The detection of HTLV infection among women reporting not having initiated sexual activity, suggests that mother-to-child transmission is also present. The high prevalence found in this study along with the long duration of breastfeeding reported (mean = 15 months) could explain high rates of vertical transmission.

We also found that lower education level was associated with HTLV-1 infection. In a study in Salvador, Brazil, formal education of less than 7 years was associated with HTLV-1 infection in females [30]. A study in Iran also found a higher prevalence of HTLV-1 infection observed among illiterate people compared to participants with higher levels of education [31]. As many of the traditional practices that may have perpetuated the persistence of HTLV-1 infection within this ethnic group (shared breastfeeding, tattoos, polygyny) have decreased with westernization and with increasing access to education.

In our study, having a most recent sex partner who was younger or of the same age, and having a most recent sex partner who worked at a logging camp were both associated with HTLV-1 infection. While the first finding is intriguing, a previous study that assessed risk factors for STI among men from 10 indigenous groups in the Peruvian Amazon found that illegal logging camps were venues where heterosexual males were often exposed to unprotected sex with FSW and MSM, and therefore at higher risk for acquiring HTLV and other STI [32]. Other studies have reported the prevalence of HTLV-1 infection among women increased with a younger initiation of sexual activity, higher number of sex partners, history of an STI and higher number of different men fathering a child by the woman [33]–[35]. In our study, the age of sexual initiation and the number of partners in the past year were similar for HTLV-1 positive and negative women but partner characteristics, such as age and previous work at a logging camp, were different.

HTLV-2 infection was associated with a self-reported history of crusted scabies and HTLV-1 infection was associated with a history of tuberculosis. Crusted scabies is a rare but severe infection caused by massive infestation with *Sarcoptes scabiei* var. *hominis* [36], [37]. In a hospital study in Brazil, all patients presenting with crusted scabies tested positive for HTLV-1 [38]; we have not identified previous reports of crusted scabies in patients with HTLV-2 infection. Regarding the association between HTLV-1 and tuberculosis, a case control study in Brazil reported a statistically significant increase in the risk of tuberculosis in subjects infected with HTLV-1 [39]. HTLV-1 has also been associated with worse outcome of tuberculosis and with a mortality rate of 25% among those infected with HTLV-1, compared with 8% in those without HTLV-1 infection [40].

Although we included non-sexually active women of reproductive age, one limitation of our study is the exclusion of participants under 15 years of age that would have provided valuable data regarding vertical transmission.

In conclusion, HTLV infection is endemic among Shipibo-Konibo women, particularly among older and less educated women. Remarkably, two characteristics of the sexual partner (younger age and labor history) were associated with infection in women. These results suggest the need for implementation of both HTLV screening during the antenatal healthcare visits of Shipibo-Konibo women, as well as counseling about the risk of HTLV transmission through prolonged breastfeeding in women infected with the virus. We also suggest the implementation of programs oriented towards preventing sexual transmission of this virus through educational interventions that cover safe-sex behavior and condom use. Additionally, we suggest clinical follow-up of HTLV infected people in order to provide early diagnosis and treatment of HTLV-associated diseases. For an intervention to be successful and tailored to this indigenous community, researchers should integrate input from the Shipibo-Konibo community into the development of evidence-based interventions.
